# Double matrix completion for circRNA-disease association prediction

**DOI:** 10.1186/s12859-021-04231-3

**Published:** 2021-06-08

**Authors:** Zong-Lan Zuo, Rui-Fen Cao, Pi-Jing Wei, Jun-Feng Xia, Chun-Hou Zheng

**Affiliations:** 1 Key Lab of Intelligent Computing and Signal Processing of Ministry of Education, School of Computer Science and Technology, Anhui University, Hefei, China; 2 Institute of Physical Science and Information Technology, Anhui University, Hefei, China; 3 Engineering Research Center of Big Data Application in Private Health Medicine, Fujian Province University, Putian, Fujian China

**Keywords:** circRNA-disease associations, Similarity matrix, Matrix completion

## Abstract

**Background:**

Circular RNAs (circRNAs) are a class of single-stranded RNA molecules with a closed-loop structure. A growing body of research has shown that circRNAs are closely related to the development of diseases. Because biological experiments to verify circRNA-disease associations are time-consuming and wasteful of resources, it is necessary to propose a reliable computational method to predict the potential candidate circRNA-disease associations for biological experiments to make them more efficient.

**Results:**

In this paper, we propose a double matrix completion method (DMCCDA) for predicting potential circRNA-disease associations. First, we constructed a similarity matrix of circRNA and disease according to circRNA sequence information and semantic disease information. We also built a Gauss interaction profile similarity matrix for circRNA and disease based on experimentally verified circRNA-disease associations. Then, the corresponding circRNA sequence similarity and semantic similarity of disease are used to update the association matrix from the perspective of circRNA and disease, respectively, by matrix multiplication. Finally, from the perspective of circRNA and disease, matrix completion is used to update the matrix block, which is formed by splicing the association matrix obtained in the previous step with the corresponding Gaussian similarity matrix. Compared with other approaches, the model of DMCCDA has a relatively good result in leave-one-out cross-validation and five-fold cross-validation. Additionally, the results of the case studies illustrate the effectiveness of the DMCCDA model.

**Conclusion:**

The results show that our method works well for recommending the potential circRNAs for a disease for biological experiments.

## Background

CircRNA is a circular single-stranded RNA molecule. Because circRNA does not have a free 5′ terminal cap and 3′ terminal tail, it is biologically stable and has a longer half-life than linear RNA molecules. It also has evolutionary conservatism and tissue specificity [1]. Existing studies have shown that circRNAs are enriched in exosomes [2], which means that they can be promising biomarkers in diagnosing disease. Identifying potential circRNA-disease associations can help understand the pathogenesis of disease at the molecular level and help identify biomarkers for diagnosing and treating disease [3].

However, biological experiments to confirm circRNA-disease associations are time-consuming and wasteful of resources. Therefore, it is urgent to predict the potential circRNA-disease associations for biological experiments to make them more efficient. In recent years, many circRNA-disease interaction databases have been built whose data are manually curated from the publications, such as CircR2Disease [4], Circ2Disease [5], and circRNADisease [6] databases. The information on these verified circRNA-disease interactions provides us with an opportunity to develop computational methods to predict potential circRNA-disease associations. Until now, much effort has been made to combine available data with different methods to predict potential circRNA-disease associations. These methods can be broadly divided into two categories; the first is the network-based method, and the second is the machine learning-based method. The network-based method usually uses a network to obtain the final prediction result. In contrast, the machine learning method usually starts with a training set of balanced positive and negative samples. Then the features and labels of the training set are used to train the model and then use the prediction model. For example, Lei et al. [7] proposed a path-weighted method to predict the circRNA-disease association based on a heterogeneous network composed of a circRNA similarity network, a disease similarity network and a circRNA-disease association network. Fan et al. [8] introduced a KATZ method for predicting the potential circRNA-disease association based on the expression profile similarity of circRNAs, the phenotypic similarity of diseases, and known circRNAs-disease associations. Wei and Liu [9] proposed an iCircDA-MF method that uses MF to predict all unknown associations. Yan et al. [10] designed a DWNN-RLS model for predicting circRNA-disease associations by using the Kronecker product kernel, which is based on the regularized least-squares method. Li et al. [11] utilized the NCPCDA method to identify potential circRNA-disease associations using multi-view similarity data, including circRNA functional similarity, disease semantic similarity, and association profile similarity. Lei and Fang [12] proposed a gradient boosting decision tree algorithm to make the final prediction using multiple biological data on circRNAs and diseases. Wang et al. [13] developed a method in which a numerical descriptor was constructed according to the similarity of diseases and circRNAs, and a deep learning convolutional neural network algorithm was used to extract the deep features of circRNA-disease descriptors. Finally, an extreme learning machine was used as the final classifier. Lei and Bian [14] proposed an RWRKNN model, where the random walk algorithm with restart is used to weight the characteristics of circRNA and the disease, and KNN was used to make the final prediction. Wang et al. [15] constructed a model named GCNCDA, which extracts features by using the graph convolutional neural network and predicts the potential circRNA-disease associations by forest penalizing attributes (Forest PA) classifier. Wang et al. [16] used a deep generative adversarial network to draw features from multi-source fusion information. They employed a logistic model tree classifier to infer the potential circRNA-disease association. Xiao et al. [17] exploited graph regularization and mixed-norm constraint terms to improve their model prediction potential for circRNA-disease associations. Li et al. [18] proposed a method to predict potential circRNA-disease associations by inductive matrix completion based on the sequence similarity of circRNAs and semantic similarity of diseases. Zhao et al. [19] developed a method that integrates the KATZ approach and the bipartite network projection algorithm to perform the prediction. Xiao et al. [20] proposed a weighted low-rank approximation optimization algorithm that combined dual-manifold regularizations to predict the potential circRNA-disease association. Ge et al. [21] exploited locality-constrained linear coding to reconstruct similarity networks and developed a label propagation method to obtain the final score matrices. Moreover, efficient association prediction models for miRNA-disease, lncRNA-disease, drug-disease, and lncRNA-miRNA are all very helpful in the design of our models and the results analysis [22–28].

Because the machine learning-based method requires reliable negative samples to train the model while there are no reliable samples, we chose the network-based method to build the model. Additionally, a model that can accurately predict is crucial for the model itself construction algorithm and essential for the selection of the dataset and its characteristics. Therefore, in this paper, we presented a double matrix completion for predicting the circRNA-disease association (DMCCDA) method, a network-based method for circRNA-disease association prediction. First, we construct a two-layer network based on known circRNA-disease associations, so the association matrix was established. Additionally, we established a Gaussian similarity matrix for circRNA and disease, respectively, according to the association matrix. Second, we construct a sequence similarity network of circRNA and a semantic similarity network of diseases, and then the corresponding similarity matrix is established. Third, we used matrix multiplication to update the association matrix from the circRNA and disease aspects. Fourth, we use double matrix completion to update the matrix block, which is composed of the updated association matrix and the corresponding Gaussian similarity matrix from two aspects. Finally, we integrated the results as the final prediction score. After we have the final model, we use leave-one-out cross-validation (LOOCV) and five-fold cross-validation (FFCV) to evaluate the performance of the DMCCDA. The value of AUC was 0.9597 under LOOCV, the mean AUC was 0.9623, and the standard deviation was 0.0029 under 100 FFCV. A case study also demonstrated that DMCCDA could accurately predict potential circRNA-disease associations.

## Result

### Performance evaluation

**Fig. 1:**
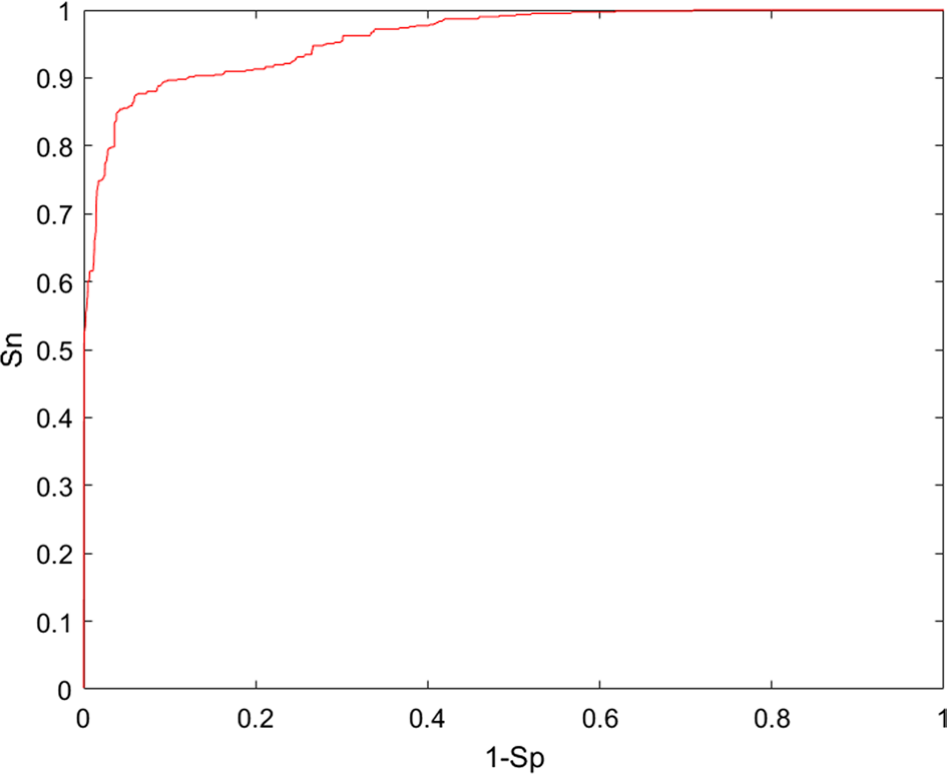
ROC of LOOCV and the value of AUC is 0.9494

In this study, to evaluate the performance of the model, we conducted global LOOCV and FFCV. The AUC value of LOOCV reached 0.9494, whereas the average AUC of 100 FFCV was 0.9623. First, all known circRNA-disease associations were treated as positive samples, and the other samples were considered candidate samples. In LOOCV, a known circRNA-disease association serves as a test sample. The remaining positive samples are used to train the model; then, we integrate the predicted scores of all the candidate samples with the predicted scores of each test sample. Finally, we drew a receiver-operating characteristic (ROC) curve and calculated the area under the ROC curve (AUC) based on the labels and the predicted scores. The corresponding ROC curves are shown in Fig. 1.

As for FFCV, we divide all known circRNA-disease samples into five parts; each part is considered as a test sample, whereas the other four parts serve as training sets, and we can integrate all known circRNA-disease sample prediction scores with all candidate samples' prediction scores and draw a ROC curve and calculate the AUC. To avoid the impact of positive sample partitioning on the performance of the evaluation model, we performed FFCV 100 times, each time selecting a different partition. Before we draw the ROC curve, we first rank the scores in descending order and set one score at a time as a threshold. If the score is greater than the threshold, the prediction is positive; if the score is less than the threshold, the prediction is negative. The true positive rate (TPR/sensitivity) and false-positive rate (FPR/1- specificity) were calculated at different thresholds. Sensitivity means that the prediction of the sample is positive, and the actual label is true. In contrast, specificity means that the prediction of the sample is positive, and the actual label is false. The true positive and false-positive rates were formulated as follows:

1 $$TPR = \frac{TP}{{TP + FN}}$$

2 $$FPR = \frac{FP}{{FP + TN}}$$

where TP indicates that the actual label of a sample is positive and the predicted result is positive, FP represents that the actual label of a sample is negative and the predicted result is positive, TN denotes that the actual label of a sample is positive and the predicted result is negative, and FN indicates that the actual label of a sample is positive, and the predicted result is negative.

### Parameter analysis

**Fig. 2:**
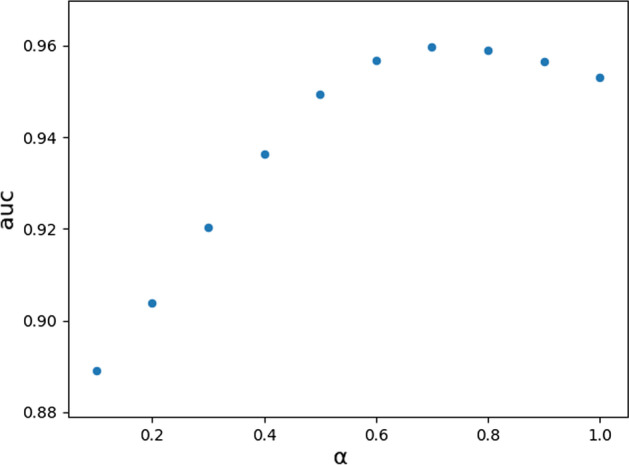
AUC values of LOOCV for different parameters

In the last step of predicting for all samples, we need to integrate two score matrices. To further improve the performance of the model, we adjusted the parameter *α* to integrate the two score matrices. We look for the most appropriate parameter *α* in the interval from 0 to 1 by setting the step size to 0.1 and calculating the AUC under LOOCV. Finally, we obtained different AUCs under different values of *α*; when the value of *α* was set to 0.7, the value of AUC under LOOCV reached the highest value of 0.9597. Therefore, we chose 0.7 as the final value of *α.* The values of AUC under LOOCV with different values of *α* are shown in the scatter diagram in Fig. 2. From Fig. 2, we can see that the values show a trend and get the highest value when *α* is 0.7, which means that the model's performance is better when we pay more attention to the scoring matrix of the circRNA space.

### The importance of model components

We conducted the following two experiments to show that matrix multiplication and double matrix completion in the model are important for predicting the potential circRNA-disease associations. On the one hand, we exploit matrix multiplication to obtain the updated association matrix from the disease perspective and circRNA perspective and integrate the matrices as the final prediction score. The AUC value of this model for LOOCV was 0.8914. On the other hand, we use double matrix completion to update the matrix block, composed of an association matrix and a Gaussian matrix. We then integrate the two new association matrices as the final prediction score, whereas the matrix multiplication operation does not update the association matrix. The corresponding AUC was 0.7811 under the LOOCV. As shown in the two experiments above, when only part of the model is used to predict the potential correlation, the results are not competitive. Therefore, we conclude that both parts of the DMCCDA model are essential for predicting potential associations.

### Prediction of a new node

**Fig. 3:**
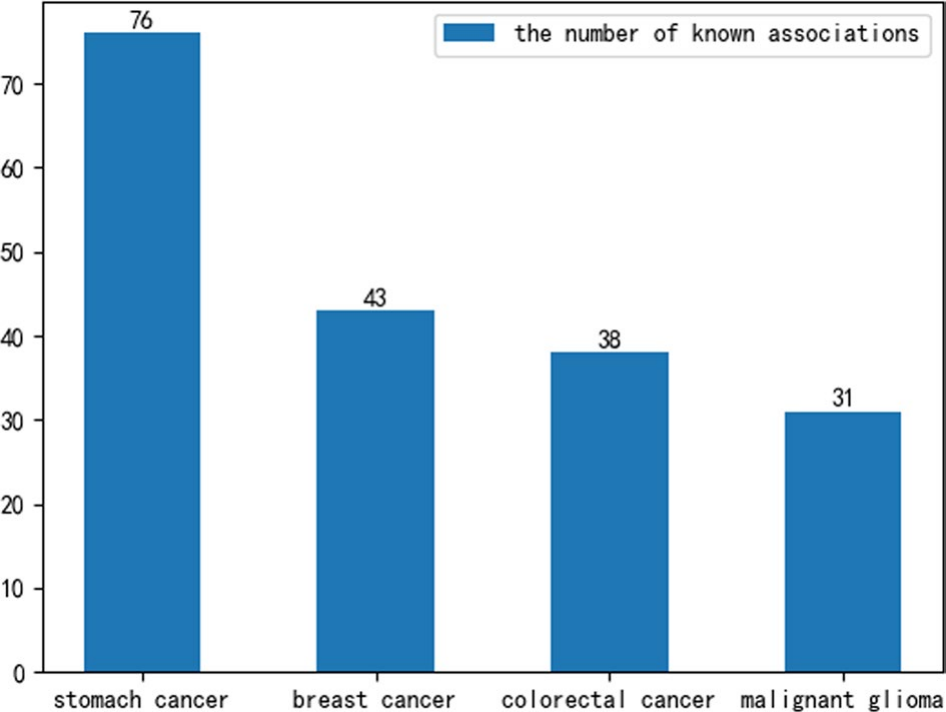
Number of known related circRNAs of four diseases

**Fig. 4:**
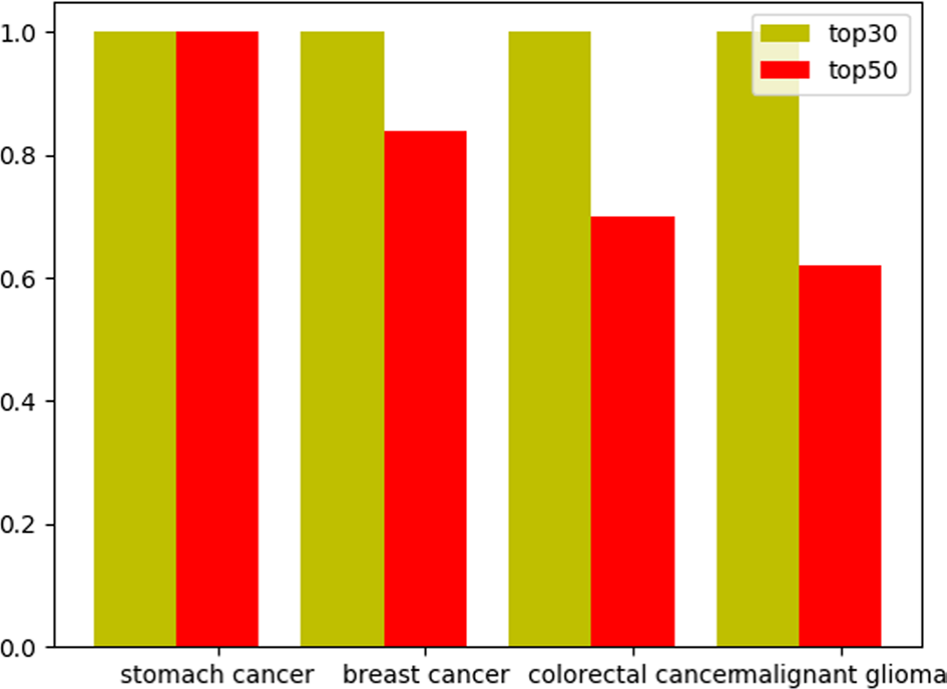
Total number of experimentally validated associations among the top 30 and top 50 of the predicted results for the four diseases

A new node in the association network is a disease node with no known circRNA associated with the disease or a circRNA node with no known disease associated with this circRNA. To evaluate the performance of our model for predicting new nodes, we selected four diseases: stomach cancer, breast cancer, colorectal cancer, and malignant glioma cancer. The total number of circRNAs known to be associated with these three diseases was the highest. Additionally, the total number of circRNAs known to be associated with them was 76, 43, 38, 31, as shown in Fig. 3. Firstly, we suppose all associations between all circRNAs, and stomach cancer are unknown. Thus, we set all values of the column corresponding to stomach cancer in the association matrix to 0. Then, we calculated the Gaussian similarity matrix according to the new association matrix and exploited the DMCCDA model to predict all samples, including associations between all circRNAs and stomach cancer. Finally, all circRNAs and stomach cancer prediction scores were ranked in descending order. We also calculated the number of associations among the top 50 predicted outcomes as known associations. As a result, we find that the top 50 predicted associations are known. We also used the same method to evaluate the performance of our model for predicting breast cancer, colorectal cancer, and malignant glioma. As for breast cancer, 43 of the top 50 predicted associations are known, 35 of the top 50 predicted associations are known for colorectal cancer, and 28 of the top 50 predicted associations are known. We also look at the top 30, and the results are shown in Fig. 4.

As shown in Fig. 3, the number of circRNAs related to these four diseases showed a decreasing trend. The total number of experimentally validated associations among the top 50 predicted results for the four diseases also decreased (Fig. 4). However, the total number of experimentally validated associations among the top 30 predicted results for the four diseases all reached 30, which means that the DMCCDA model has high efficiency in predicting potential circRNA-disease associations.

### The effect of the model on different datasets

We have shown above that our known circRNA-disease associations were collected from three databases. We built the association matrix after integrating the data, built the corresponding association matrix for the associated data in the three databases, and used our model to make predictions for the samples involved in the three association matrices. Data in the database CircR2Disease were processed into 445 known association pairs, involving 389 circRNAs and 61 diseases, and data in the database Circ2Disease were processed into 240 known associations, involving 215 circRNA and 46 diseases. The circRNADisease database was processed into 241 known associations, involving 223 circRNAs and 34 diseases. The LOOCV was used to evaluate the performance of our model on these datasets, and the AUCs were 0.9673, 0.9447, and 0.9568, respectively. From the high value of the AUC, we can conclude that our model can obtain good results on different datasets.

### Compare with other methods

To prove the effectiveness of our method, we compared it with five state-of-the-art methods, that is, NCPCDA [11], PWCDA [7], iCircDA-MF [9], RWRKNN [14], and GCNCDA [15]. Among them, three methods (NCPCDA, PWCDA, and iCircDA-MF) are network-based approaches, and the rest are machine learning-based methods. We compared our DMCCDA method with these five methods in terms of the AUC under FFCV. The corresponding AUC values of all these methods are listed in Table 1. We can see that the AUC value in our method reached 0.9623, whereas the AUC values of NCPCDA, PWCDA, iCircDA-MF, RWRKNN, and GCNCDA were 0.9201, 0.890, 0.9178, 0.9333, and 0.9090, respectively. Therefore, we can conclude that our method is superior to other methods, and our method can be used to predict potential circRNA-disease associations.

**Table 1:** AUC values of different models under FFCV

Methods	NCPCDA	PWCDA	iCircDA-MF	RWRKNN	GCNCDA	DMCCDA
Auc(FFCV)	0.9201	0.8900	0.9178	0.9333	0.9090	0.9623

### Case study

To further verify the efficiency of our model, we selected the top ten samples from all unknown circRNA-disease pairs according to the score produced by our model. We used the published literature to verify the prediction results (Table 2). We can see that six of the top 10 prediction samples are verified in the literature. Based on the existing literature, we can verify that circRNA hsa_circ_0001649 and circRNA hsa_circ_0001141 are related to liver cancer [29, 30]. In addition, a recently published paper showed that circRNA hsa_circ_0000284 promotes gemcitabine sensitivity in bladder cancer [31] and has also been verified to be related to esophageal cancer via microRNA [32]. A published study also showed that circRNA hsa_circ_0001141 is associated with esophageal cancer [33]. Another study showed that circRNA has_circ_0001141 inhibits stomach cancer migration, invasion, and proliferation by regulating the Wnt/β-catenin pathway [34].

**Table 2:** Top 10 circRNA-disease associations predicted by our model

Rank	Diseases	circRNAs	PMID
1	Liver cancer	hsa_circ_0001649	28185365
2	Liver cancer	hsa_circ_0001141	29760792
3	Bladder carcinoma	hsa_circ_0000284	32194801
4	Hepatoblastoma	hsa_circ_0000284	Unconfirmed
5	Esophageal cancer	hsa_circ_0000284	32189968
6	Esophageal cancer	hsa_circ_0001141	28969099
7	Pharynx squamous cell carcinoma	hsa_circ_0000284	Unconfirmed
8	Stomach cancer	hsa_circ_0001141	33060778
9	Triple-receptor negative breast cancer	hsa_circ_0000284	Unconfirmed
10	Esophageal cancer	hsa_circ_0001649	Unconfirmed

## Conclusion

Identifying circRNAs associated with the disease can provide a better understanding of the pathogenesis of the disease at the molecular level and help identify biomarkers of the disease and the design of drugs. In this paper, we propose a novel method, DMCCDA, to predict potential circRNA-disease associations for biological experiments to promote its efficiency and reduce resource consumption. First, we integrated circRNA-disease associations from three databases collected by circRNA-disease associations manually from published papers and constructed an association matrix to obtain as many experimentally verified circRNA-disease associations as we can. Second, we calculated the circRNA sequence similarity based on the circRNA sequence information and disease semantic similarity according to the disease ontology. Additionally, we calculated the Gaussian similarity matrices according to the association matrix for circRNA and disease. Finally, we exploit the matrix multiplication operation to update the association matrix from circRNA and disease respect by circRNA sequence similarity and disease semantic similarity. Then, we used matrix completion to predict all the unknown samples. We mainly evaluated the performance of the model using the AUC of LOOCV and FFCV. The experimental results and case study demonstrate the high efficiency of the model.

Although many models have been developed to predict potential circRNA-disease associations, there are still many problems in this field. For instance, we lack reliable negative samples to develop machine learning-based methods. Additionally, some models cannot make predictions for new nodes, such as a new disease that has no known related circRNAs or a circRNA that has no known related diseases. In this study, we developed a semi-supervised method to predict potential circRNA-disease associations, which means that we do not need negative samples. Additionally, our model can be used to predict the new nodes. However, our model had some limitations. First, the method used to calculate the similarity between circRNAs and diseases is insufficient. Second, matrix completion is often used to complete the missing values. However, in this study, matrix completion is used to update the new association matrices that integrate the similarity in circRNA and disease spaces, which may introduce some noise. In the future, we will consider these problems and design a better method to predict potential circRNA-disease associations. In addition, as the association prediction, the circRNA-disease association prediction has a close relationship with the microRNA-disease association prediction, the lncRNA-disease association prediction, and drug reposition. We will pay attention to these kinds of association prediction, which belong to association prediction and are essential for diagnosing and treating complex human diseases. As for these types of association prediction methods, there are many innovative methods based on deep learning to learn the feature representation and achieve a good result. In the future, we will design a model that considers the characteristics and reduces the impact of unverified negative samples on the model.

## Methods

### Human circRNA-disease associations

To make full use of the information available, we collected experimentally verified associations from three databases, including CircR2Disease, Circ2Disease, and circRNADisease. All–RNA the disease associations of these three databases were manually collected from published articles. The CircR2Disease database collected 739 circRNA-disease associations involving 512 circRNAs and 71 diseases from articles published before March 31, 2018. The Circ2Disease database collected 273 circRNA-disease associations involving 237 circRNAs and 54 diseases from articles published before November 1, 2017. The circRNADisease database collected 354 circRNA-disease associations involving 330 circRNAs and 48 diseases from articles published before November 2017. First, we extracted human circRNA-disease associations, integrated circRNA-disease associations from three databases and removed duplicate associations. Then, we deleted a part of these circRNA-disease associations that include the circRNA, which has no circRNA sequence information in circBase or contains the disease that has no disease ontology identity (DOID) information in the Disease Ontology (DO) database [35]. Finally, we obtained 609 circRNA-disease associations involving 512 circRNAs and 71 diseases. Additionally, we constructed an adjacency matrix A that has *nc* rows and *nd* columns, whereas *nc* represents the total number of circRNAs, and *nd* denotes the total number of diseases that are involved in the known circRNA-disease associations. If a certain circRNA is experimentally verified to be related to a certain disease, the element in the corresponding position in matrix A is 1; otherwise, it is 0.

### circRNA sequence similarity

To calculate circRNA sequence similarity, we first downloaded 140,790 circRNA sequence information from the database circBase [36] and then extracted the circRNA sequence information from the known associations involved. Next, we utilized the Levenshtein distance [18] to measure the similarity between any two circRNAs. The Levenshtein distance represents the minimum number of operands required to convert string A sequence to string B, which means that the shorter the distance, the greater the similarity between the two circRNAs. Formula (3) was used to calculate the sequence similarity between the two circRNAs.

3 $$SimC(circ_{i} ,circ_{j} ) = 1 - \frac{{dis(circ_{i} ,circ_{j} )}}{{len(circ_{i} ) + len(circ_{j} )}}$$

where *dis* describes the number of operands needed to convert circRNA *circ*_*i*_ sequence to circRNA *circ*_*j*_ sequence, and *len* represents the sequence length of some circRNAs.

### Disease semantic similarity

As for disease semantic similarity, we first collected disease DOID information from the database Disease Ontology. Then we used the DOSim [37] function to calculate disease semantic similarity based on Wang's method [38]. Because there is an R package DOSE, we can easily obtain the disease semantic similarity by inputting the disease DOID. Wang's method was based on the following formula:

4 $$S{\text{im}}D(d_{i} ,d_{j} ) = \frac{{\sum {_{{t \in T_{{d_{i} }} \cap T_{{d_{j} }} }} } (S_{{d_{i} }} (t) + S_{{d_{j} }} (t))}}{{\sum {_{{t \in T_{{d_{i} }} }} S_{{d_{i} }} (t) + \sum {_{{t \in T_{{d_{j} }} }} S_{{d_{j} }} } (t)} }}$$

where *Tdi* represents disease *di* and all ancestor node of disease *di* in the directed acyclic graph of disease, and *SDI *(*t*) indicates the contribution from all nodes in the set *Tdi* to disease *di*. The details are shown in the following formula:

5 $$\left\{ {\begin{array}{*{20}l} {S_{{d_{i} }} (d_{i} ) = 1} \hfill \\ {S_{{d_{i} }} (t) = \max \{ w_{e} *S_{{d_{i} }} (t^{\prime})|t^{\prime} \in childrenof(t)\} } \hfill \\ \end{array} } \right.$$

### Gauss interaction profile kernel similarity

The Gaussian interaction profile kernel similarity is another algorithm constructed to measure disease similarity and circRNA similarity based on the known association matrix. As for the association matrix, the *ith* row *IP *(*i*) represents the associations between the *ith* circRNA and all diseases. The *jth* column *IP *(*j*) denotes the associations between the *jth* disease and all circRNAs. Based on the assumption that similar circRNAs are more likely to be associated with similar diseases and vice versa, we calculated the Gaussian interaction profile kernel similarity for circRNA and disease as follows:

6 $$KD{\text{(d}}_{{\text{i}}} {\text{,d}}_{{\text{j}}} {)} = \exp ( - \beta_{d} ||IP({\text{d}}_{{\text{i}}} ) - IP({\text{d}}_{{\text{j}}} )||^{2} )$$

7 $$KC({\text{c}}_{{\text{i}}} ,{\text{c}}_{{\text{j}}} ) = \exp ( -\upbeta _{c} ||IP({\text{c}}_{{\text{i}}} ) - IP(c_{{\text{j}}} )||^{2} )$$

where *β*_*d*_ and *β*_*c*_ are the kernel bandwidths, which can be calculated as follows:

8 $$\beta_{{\text{d}}} = \beta ^{\prime}_{{\text{d}}} /(\frac{1}{nd}\sum\nolimits_{i = 1}^{n} {||IP(d_{i} )} ||^{2} )$$

9 $$\beta_{c} = \beta_{c}^{^{\prime}} /\left( {\frac{1}{nc}\sum\nolimits_{i = 1}^{c} {||IP(c_{i} )||^{2} } } \right)$$

where *β*_*d*_′ and *β*_*c*_′ are the original bandwidths, and according to previous research, we assign the value of the initial bandwidth to 1. Finally, we can obtain the Gaussian interaction profile kernel similarity matrix KD, KC for disease, and circRNA, respectively.

### Model construction

To make full use of the known information and find the potential associations, we use the following steps to predict potential circRNA-disease associations in this paper. First, we collected the known circRNA-disease associations, sequence information about circRNAs, and semantic information about diseases. Second, we construct association matrix A, sequence similarity matrix CC, and semantic similarity matrix DD based on the information gathered in the step above.

**Fig. 5:**
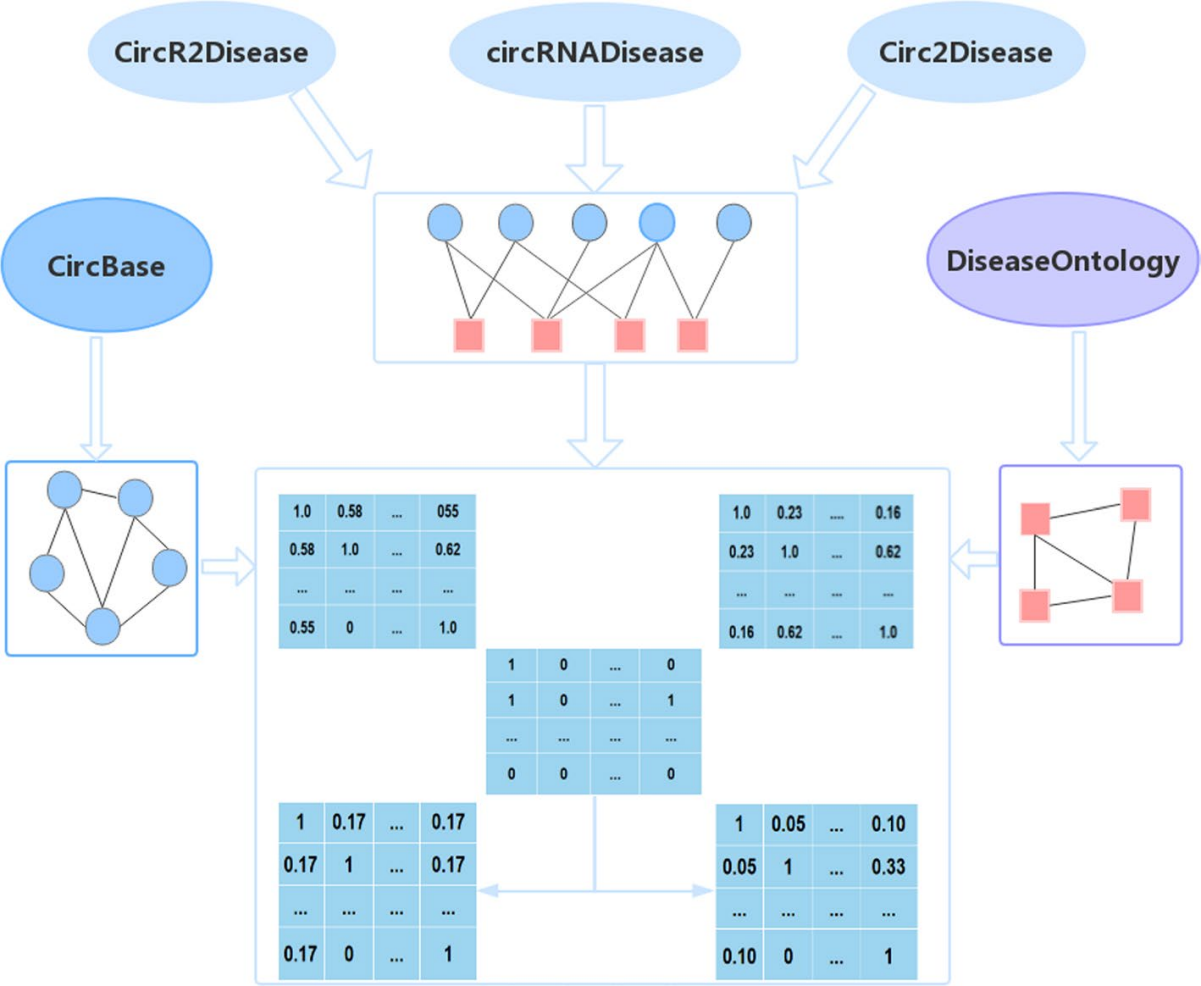
Preparation materials mainly include association matrix, semantic similarity matrix of disease, sequence similarity matrix of circRNA, and Gaussian similarity between diseases and between circRNAs

**Fig. 6:**
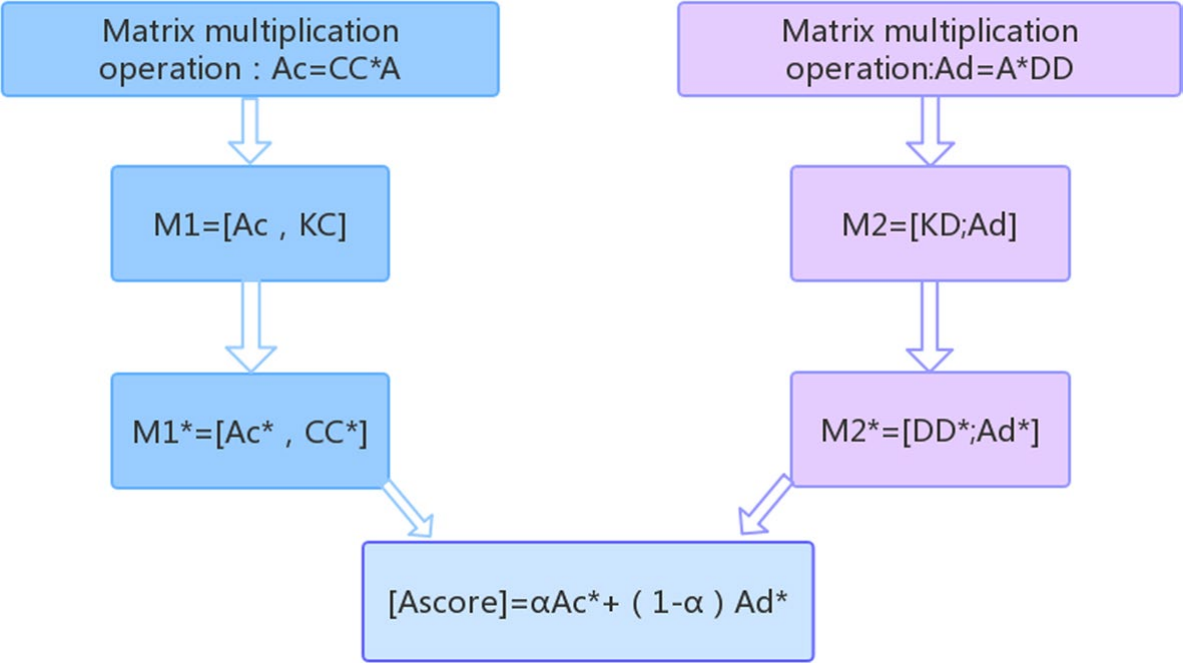
Flowchart of prediction, step 1: matrix multiplication to update the association matrix by similarity matrix. step 2: constructing a matrix block. step 3: matrix completion to update the matrix block. Step 4: integrating the scoring matrix of disease and circRNA space

Additionally, we calculated the Gaussian similarity matrix KC and KD for circRNA and disease according to the association matrix. The preparation materials are shown in Fig. 5. Third, we exploit the similarity of circRNA and diseases based on the sequence and semantic information of circRNAs and diseases to update the association matrix and obtain two updated association matrices Ac and Ad. Fourth, we use double matrix completion to update the matrix block, which is composed of the updated association matrix and the corresponding Gaussian similarity matrix from two aspects. Finally, we extract the association matrix parts Ac* and Ad* from the two matrix blocks after using the matrix completion algorithm and integrated the two matrices as the predicted score of each sample. The corresponding flowchart is shown in Fig. 6, in which we introduce the association matrix (A), sequence similarity matrix (CC), and Gaussian similarity matrix (KC) of circRNA, and introduced the semantic similarity matrix (DD) and Gaussian similarity matrix (KD) of the disease. Then, we use matrix multiplication to update the association matrix; thus, even if we obtain a new disease with no known circRNA association, we can predict this disease based on the semantic similarity between this disease and other diseases. The formulae are as follows:

10 $$A{\text{c}} = CC*A$$

11 $$Ad = A*DD$$

To control the values of Ac and Ad within a specific range, we carried out the following processing for Ac and Ad [11].

12 $$Ac(i,j) = \frac{Ac(i,j)}{{A(:,j)}}$$

13 $$Ad(i,j) = \frac{Ad(i,j)}{{A(i,:)}}$$

Then, we can obtain two updated association matrices Ac and Ad, which integrate the similarity of circRNAs and diseases, respectively. Next, we splice the updated matrix Ac and the Gaussian matrix KC to form the first matrix block M1. Similarly, we splice Ad and the Gaussian matrix KD to form the second matrix block M2. To make the best use of the circRNA-disease association matrix information and similarity information, we used two matrix complements to update the two matrix blocks obtained in the previous step in circRNA and disease space, respectively, which was also inspired by Yang et al. [39]. First, in the circRNA space, we integrate the bounded nuclear norm regularization to the nuclear norm minimization problem [40], as follows:

14 $$\begin{aligned} & {\text{min}}||M1||_{*} + \frac{\alpha }{2}||P_{\Omega } (M1) - P_{\Omega } (M)||_{F}^{2} \\ & \quad {\text{s}}.{\text{t}}.\quad 0 \le M1 \le 1 \\ \end{aligned}$$

where ||M1||* represents the nuclear norm of M1, P is the projection operation, and Ω is the universal set. Additionally, *α* is a harmonic parameter, and the initial value of M is M1. W is a new matrix that the following formula can represent:

15 $$\begin{aligned} & {\text{min}}||M1||_{*} + \frac{\alpha }{2}||P_{\Omega } (W) - P(M)||_{F}^{2} \\ & \quad {\text{s}}.{\text{t}}.\quad M1 = W,0 \le W \le 1 \\ \end{aligned}$$

Then, we can get the model's augmented Lagrangian function as follows,

16 $$\begin{gathered} L(W,M1,Y,\alpha ,\beta ) = ||M1||_{*} + \frac{\alpha }{2}{||}P_{\Omega } (W) - P_{\Omega } (M)||_{F}^{2} \hfill \\ \quad + Tr(Y^{T} (M1 - W)) + \frac{\beta }{2}||M1 - W||_{F}^{2} \hfill \\ \end{gathered}$$

where Y is the Lagrange multiplier, and *β* is the penalty coefficient. Then, the closed result is obtained as follows:

17 $$W_{k + 1} = (L - \frac{\alpha }{\alpha + \beta }P_{\Omega } )(\frac{1}{\beta }Y_{k} + \frac{\alpha }{\beta }P_{\Omega } (M) + M1_{k} )$$

We can also get the value of M1 and Y by iterating,

18 $$M1_{k + 1} = \mathop {\arg \min }\limits_{M1} ||M1||_{*} + \frac{\beta }{2}||M1 - (W_{k + 1} - \frac{1}{\beta }Y_{k} )||_{F}^{2}$$

19 $$Y_{k + 1} = Y_{k} + \beta (M1_{k + 1} - W_{k + 1} )$$

Therefore, by iterating to convergence, we obtain the final recovery matrix W. We similarly conduct matrix M2 in the disease space, and we can obtain the updated matrix Ad*. Finally, we obtain two scoring matrices and integrate them using the following equation:

20 $${\text{Ascore}} = \upalpha {\text{Ac}}^{*} + \left( {1 - \upalpha } \right){\text{Ad}}^{*}$$

where *α* is the integration parameter. After the relevant experiments, we set the parameter value to 0.7. Then, we can obtain all the samples' scoring matrix Ascore.

## Data Availability

All data and code underlying this study are available in an online archive https://github.com/zzl1996zzl/DMC-CDA.

## Abbreviations

circRNA: Circular RNA
DMCCDA: Double matrix completion for predicting the circRNA-disease association
LOOCV: Leave-one-out cross validation
FFCV: Five-fold cross validation
DOID: Disease ontology identities
DO: Disease ontology
ROC: Receiver-operating characteristics
AUC: Area under the ROC curve
TPR: True positive rate
FPR: False positive rate
TP: True positives
FP: False positives
TN: True negatives
FN: False negatives
